# Novel interfaces for internet of wearable electrochemical sensors

**DOI:** 10.1039/d4ra07165d

**Published:** 2024-11-18

**Authors:** Suniya Shahzad, Faiza Jan Iftikhar, Afzal Shah, Hassan Abdur Rehman, Emmanuel Iwuoha

**Affiliations:** a National University of Technology (NUTECH) Islamabad 44000 Pakistan faizajiftikhar@nutech.edu.pk; b Department of Chemistry, Quaid-i-Azam University Islamabad 45320 Pakistan afzals_qau@yahoo.com; c Sensorlab, Department of Chemistry, University of the Western Cape Private Bag X17 Bellville 7535 South Africa

## Abstract

The integration of wearable devices, the Internet of Things (IoT), and advanced sensing platforms implies a significant paradigm shift in technological innovations and human interactions. The IoT technology allows continuous monitoring in real time. Thus, Internet of Wearables has made remarkable strides, especially in the field of medical monitoring. IoT-enabled wearable systems assist in early disease detection that facilitates personalized interventions and proactive healthcare management, thereby empowering individuals to take charge of their wellbeing. Until now, physical sensors have been successfully integrated into wearable devices for physical activity monitoring. However, obtaining biochemical information poses challenges in the contexts of fabrication compatibility and shorter operation lifetimes. IoT-based electrochemical wearable sensors allow real-time acquisition of data and interpretation of biomolecular information corresponding to biomarkers, viruses, bacteria and metabolites, extending the diagnostic capabilities beyond physical activity tracking. Thus, critical heath parameters such as glucose levels, blood pressure and cardiac rhythm may be monitored by these devices regardless of location and time. This work presents versatile electrochemical sensing devices across different disciplines, including but not limited to sports, safety and wellbeing by using IoT. It also discusses the detection principles for biomarkers and biofluid monitoring, and their integration into devices and advancements in sensing interfaces.

## Abstract 2

The integration of wearable devices, the Internet of Things (IoT), and advanced sensing platforms imply a significant paradigm shift in technological innovations and human interactions.

## Graphical abstract

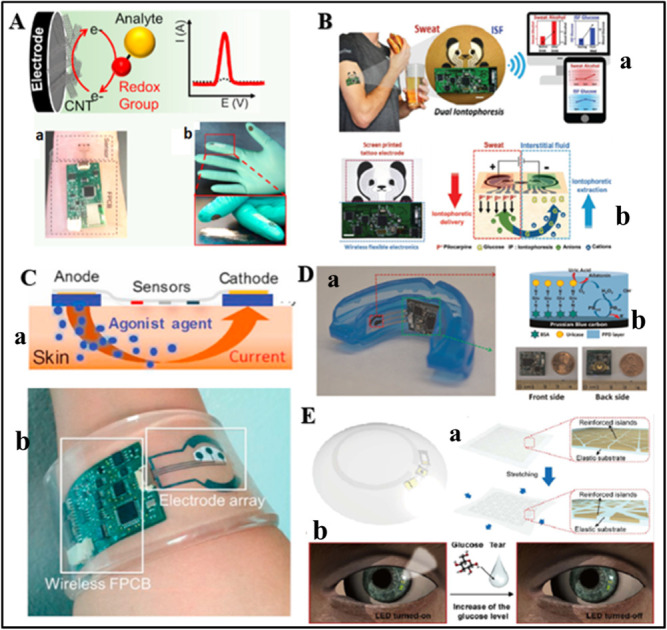

## 1. Introduction

The convergence of wearable devices, the Internet of Wearable Things and advanced sensing platforms heralds a transformative era in technology and human interactions. Wearable sensing devices have progressed in recent years and have demonstrated their potential for integrated systems that span various domains. These encompass future communications, wearable medical sensors, epidermal skin technology, smart glasses, health monitoring, microchip technology and the Internet of Things (IoT) technology. At the heart of this convergence lies the fusion of sensing devices with advanced materials. Specifically, wirelessly connected electrochemical sensors are seamlessly integrated into wearable devices, enabling robust real-time data collection and analysis of biomarkers, such as electrolytes, metabolites (such as potassium ions), proteins, hormones, as well as viruses and bacteria. Biomarkers exist in body fluids and tissues as well as in tears and sweat. They contain valuable information for diagnostics and offer insights beyond mere physical activity tracking. Biomarkers are detected by electrochemical sensors integrated with flexible substrates in the form of lenses, smart fabrics, skin patches and accessories. Hence, wearable electrochemical sensors, at the forefront of technological innovation, are not only reshaping the landscape of biomedicine, security and wellness as sensing platforms but are also poised to play a pivotal role in the emerging field of the Internet of Wearable Electrochemical Sensors connected by a web of the Internet of Things, termed IoT.^1,2^ This IoT-based technology holds immense promise in impacting many disciplines with applications ranging from health and fitness, through environmental monitoring, safety and security, to entertainment and augmented social interactions.

**Fig. 1:**
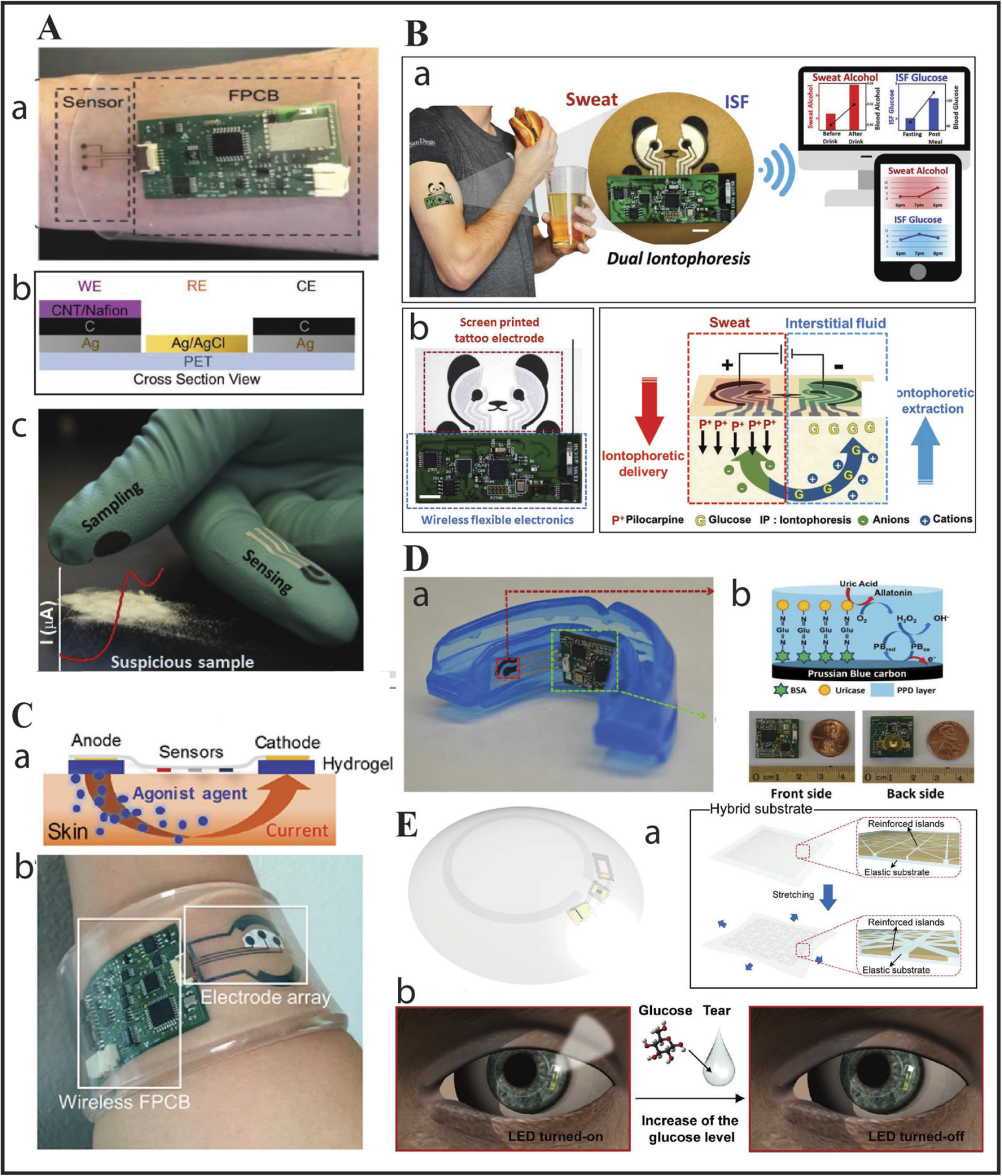
Body wearable devices: (A) voltammetric sensors: (a) as sweat patch regulating the drug methylxanthine; (b) schematic of caffeine sensing band; and (c) fentanyl detecting gloves with square-wave voltammetry. Reproduced from ref. 6 with permission from Wiley-VCH, copyright 2018 & reproduced from ref. 7 with permission from Elsevier, copyright 2019. (B) Another wearable dual iontophoretic biosensor based on a tattoo, to simultaneously detect glucose and sweat: (a) dual iontophoretic biosensor for ISF glucose and sweat alcohol detection on a human subject with data being transmitted wirelessly to a mobile device; (b) screen printed electrode with wireless circuit on which the iontophoretic operation takes place. Reproduced from ref. 8 with permission from Wiley-VCH, copyright 2018. (C) Illustration of drug delivery *via* the iontophoretic technique: (a) and (b) showcase an integrated wristband for the detection of sweat *via* iontophoretic technique (sensors based on detecting cystic fibrosis electrochemically). Reproduced from ref. 9 with permission from the National Academy of Sciences, copyright 2017. (D) (a) Biosensor as a mouthguard with wireless circuit board integrated to an amperometric genosensor for detection of UA; (b) carbon WE modified with Prussian Blue attached to Uricase *via* BSA for detection of UA in saliva and photos showing wireless circuits from front and back side. Reproduced from ref. 10 with permission from Elsevier, copyright 2015. (E) (a) Schematics of a smart contact lens composed of a hybrid substrate which is embedded with stretchable conductors and functional devices to detect glucose in human tears in real-time; (b) schematics showing working of the lens by wireless transmission of power which activates the LED pixel and thus the sensor for detecting glucose. As the glucose level in the tear reaches a threshold level the LED pixel switches off. Reproduced from ref. 11 with permission from the American Association for the Advancement of Science, copyright 2018.

Recent innovations in wearable electrochemical sensors became possible *via* a synergy of several key advancements, including proliferation of wireless connectivity, development in materials science and engineering, advancement in energy harvesting technologies and the miniaturization of sensor electronics.^3^ Development in materials and nanoscience technology has facilitated the construction of flexible electrodes and substrates for circuit printing and signal recognition *via* modern microfabrication techniques.^4,5^ Advancements in the field of IoT have enabled the unified integration of sensing, processing and feedback units, to furnish real-time, continuous monitoring and data analysis (Fig. 1). The Internet of Wearables (IoW) signifies a paradigm shift in the use of networking technology, and has significant implications in improving the quality of life.^3,12^ Thus, within the realm of connected intelligence, a significant breakthrough has emerged as a category of incredibly efficient smart wearable devices known as IoW. These devices are non-invasive, inexpensive, portable, easy to use and capable of enabling us to counter many real-life challenges.

The term IoW refers to electronic platforms designed to connect individuals with the digital world. It detects the interactions of the user with the surrounding environment, subsequently transferring the obtained data to a processing element that relays customized information to users, thereby improving their performance and overall quality of life. Multiple physical and chemical sensors are integrated together to form a full real-time picture of a person's health condition. However, despite its figures of merit, challenges persist regarding the selection of viable substrate materials for effective wearable sensors. Thus, the identification, quantification and analysis of biomarkers, as well as signal detection and sampling challenges, hinder reliable functioning of these devices. This can be addressed by improving constituents, fabrication techniques and design schemes.^13^ To mention a few examples, carbon nanotubes (CNTs) and graphene have been reported to demonstrate their suitability as ideal materials for skin-compatible electrochemical platforms of IoW sensors.^5^

Miniaturized electrochemical sensors are of special interest, as they play an essential role in IoW. These chemical sensors use the electrode–analyte interface to measure electrical properties such as current, impedance or potential, for obtaining information about the target analyte while keeping all operations and functionalities intact as the size is reduced. In essence, electrochemical systems provide information regarding chemical and biochemical systems by establishing a relationship between chemical/biological and electrical parameters. These devices may be integrated into everyday objects for the detection of various human health parameters such as blood oxygen saturation and heart rate.^14–22^ Examples of such devices include microneedle sensing platforms,^23,24^ electronic skin-based pressure sensors^25^ and *in vivo* microsystems.^26^ Karyakin and coworkers have developed a flow-through wearable biosensor based on noninvasive detection of hypoxia and diabetes in sweat.^27,28^ Similarly, Wang *et al.* highlighted the development of wearable sensing techniques in sweat for the immediate detection of alcohol without the need for performing an invasive sampling of blood. These biosensors employ two enzymes: alcohol oxidase (AOx) and alcohol dehydrogenase (ADH) to catalyze alcohol redox reactions, resulting in a substantial increase in sensitivity for alcohol detection. The detected concentrations of alcohol in sweat were in agreement with simultaneous alcohol levels examined through blood testing, thus supporting the idea of developing sweat-based wearable biosensors for the detection of alcohol.^8,29,30^ Most of the reported papers for the miniaturization of electrodes have used Whatman grade 1 filter paper, which are printed with photoresist or wax to define specific regions for the detection of electroactive products.^31^ These miniaturized microelectrodes may be integrated with microfluidic devices modified with smart materials and are promising for future IOT-based devices. Further to this discussion, organic electrochemical transistor biosensors allow the detection of both electroactive and inactive molecules *via* electrostatic interactions. These OECTs have a low working voltage and are easily fabricated. These sensors can easily be miniaturized to detect biomolecules, as they are not affected if the size of the device is changed. An OECT sensor integrated into a flexible microfluidic system that comprises a Au gate and a DNA probe with a detection limit for DNA as 10 pM is reported.^32^ Similarly a diaper embedded biosensor for detecting glucose in simulated urine was connected to a mobile device *via* Bluetooth and has been successful in collecting signals.^33^

Generally, all types of electrochemical wearable biosensor devices contain seven elements, namely a transducer that is comprised of a three-electrode system (working, counter and reference electrodes) where the surface of working electrode is immobilized by a bio-receptor, an amplifier, an electrical circuit, an analyzer, a remote transmission segment, an output device and software. The substrate part connects with a person's body, while the transducer receives signals from bio-chemical reactions triggered by the bio-receptor when it reacts with the target analyte. The amplifier circuits detect and enhance this signal from the transducer, which is further interpreted by remote transmission segment and output device. The substrate materials employed in skin sensors can be organic such as polyethylene terephthalate (PET) and polydimethyl siloxane (PDMS), economic and ecological, *i.e.* Ecoflex (a compostable plastic) and natural substances such as fabrics and cellulose paper. Natural substances are incorporated when sensors are needed to overlay on clothes rather than the skin.^34^

The principal advantages of different types of wearable sensors working in conjunction with wireless communication include portability and real-time data acquisition. However, the compatibility of materials and substrates for construction and sensing is the main challenge associated with this technology. For instance, complementary metal–oxide–semiconductor (CMOS) interfaces have been reported to be compatible when used in physical sensors. However, chemical sensors are usually incompatible with CMOS and many other microelectronic processes, necessitating frequent replacement or calibration.^35,36^ Thus, there is a pressing need to address the challenges emerging in the realm of chemical sensing, especially electrochemical sensing, which is the main subject of discussion in this review. Moreover, the current review specifically uncovers IoW as portable devices for electrochemical sensing in various fields of life, such as health, sport, safety at work, social interactions, military, and beyond. The focal point of this article is to present the key challenges emerging in the realm of chemical sensing, especially electrochemical sensing, as well as providing potential solutions specifically *via* utilization of advanced materials for the design of novel sensing interfaces.

## 2. Applications of wearable sensors

The applications of wearable electrochemical sensors mostly revolve around healthcare, diagnosis and fitness. However, there is growing interest in areas such as safety and security, defense and hazard mitigation, quality analysis of food and the environment, entertainment and social networking, and augmented learning. Wearable sensors lately attracted considerable attention in applications regarding fitness and healthcare since they offer instantaneous information about health performance of the wearer. Perpetual monitoring of chemical parameters is significant as it helps in the prediction of impending health risks. Several research groups have carried out advanced non-invasive innovations for monitoring biomarkers such as metabolites and heavy metals in biofluids (for example, in saliva).^37^ Such biofluids can be potentially used to create a pain-free route for monitoring biomarkers without blood samples, allowing immediate tracing of analytes. There have been review articles based on wearable electrochemical sensors describing the detection of mycotoxins,^38^ porphyrin and graphene-based sensors,^39^ the detection of plant molecules,^40^ and molecular imprinted polymer-based sensors for the detection of pesticides.^41^ Additionally, there are articles on wearable device sensors based on biomarkers for health monitoring. However, these articles focus on only one aspect of the topic. In contrast, this review comprehensively tries to capture novel electrochemical interfaces specifically used in IOT integrable wearable devices, which have not been previously focused on previously, while emphasizing its applications. The following are some applications of electrochemical sensors to detect biomarkers of significance that play a crucial role in healthcare and diagnosis while the ongoing research aims to convert them into wearable devices.

### 2.1. Healthcare and diagnosis

The most important application of health-related wearable chemical sensors is to aid in early detection and prevention of various lifestyle-related ailments such as cardiovascular diseases and diabetes mellitus.^42,43^ Blood glucose is usually quantified using colorimetric or amperometric biosensors, which employ dehydrogenase or oxidase enzymes to facilitate the measurement of glucose oxidation.^44^ Long-term stability issues associated with such enzyme-based systems have prompted researchers to conduct active research on enzyme-free glucose sensors. Despite this, glucose monitors have advanced significantly over time.^45^ For instance, they can detect blood glucose *via* interstitial fluids within a two-week timeframe,^46^ signifying a marked improvement in the quality of life for diabetic patients. As the saying goes a sound body portrays a sound mind, which makes fitness and sports the utmost priority for everyone with a need to measure physical fitness *via* wearables. The main goal of sports-related wearables is to optimize athletic performance by enabling the early detection or prevention of injuries. These devices use sweat as a real-time medium to monitor the exertion levels and hydration status during physical activities, thus aiding in maintaining peak performance.^47,48^ Consequently, effective management of sweat measurements necessitates the implementation of smart strategies. Some approaches use microfluidics,^49–51^ while others rely on lateral flow membranes or absorbents.^52^ Early diagnosis of biomarkers such as cytokines and creatine kinase indicative of muscle damage is essential to prevent injuries in professional sports. For example, label-free monitoring of interleukin-6 (IL-6) has been reported to measure artificial sweat *via* electrochemical impedance spectroscopy.^53^ Furthermore, monitoring dehydration is crucial in sports as even a dehydration level exceeding 2% of an individual's net body weight can lead to a significant decline in sports performance.^54^ Additionally, lactate and glucose can be measured in sweat using amperometric biosensors, where sweat lactate serves as an indirect indicator of physical or sweat-based gland activity.^55,56^

### 2.2. Environmental analysis

In the era of severe climate change, environmental sensing systems coupled with wearing devices allow the monitoring of CO_2_ emissions and detect a safe operating space. Moreover, these systems are capable of identifying hazardous environments containing volatile organic compounds, toxic gases and industrial vapors enhancing safety and environmental awareness. Wearable sensing devices have also been shown to provide early warnings regarding possible COVID-19 contamination, detecting symptoms before serious illness sets in. Additionally, real-time virus detection has been achieved using metal–organic frameworks or MOF. User-readable Radio Frequency Identification (RFID) tags integrated with smart bandages have been reported for wearable electrochemical sensor applications. Furthermore, ultra-wide band (UWB) radio tags with incorporated multiwall carbon nanotubes (MWCNTs) combined with a conductometric Cu electrode enable the detection of nitrogen dioxide levels in the environment.

In a previous report, an enhanced laser-induced graphene (LIG) printing method has been used to fabricate a sensor that can detect within 15.6 seconds with six sensors in a single set. It is a lightweight sensor, which can be linked to any portable electrochemical workstation or smartphone for real-time detection of salicylic acid, pH and neonicotinoid insecticides.^57^ In another work, a glove-based wearable sensor has been reported for the investigation of perilous substances such as 4-nitrophenol and picric acid. This electrochemical sensor offers real-time monitoring of contaminants assisting in public safety and a clean environment.^58^ Zhang, Qing, *et al.* reported the use of smart technology-based wearable sensors in plants for the detection of an organo-phosphorus pesticide known as methyl parathion, which is harmful for human health if in excess concentrations. This microfluidic sensor was constructed with LIG technology, which offers excellent LOD.^59^

### 2.3. Security and defense

In the realm of security and defense, technologies mirroring those utilized in sports-related wearables are often employed despite the stringent demands for reliability and robustness. For instance, smart garments and fingertip sensing devices with integrated electrochemical sensors can be used for the determination of nerve agents, nitroaromatic explosives, gunshot residues, and security threats in marine environment, highlighting their critical role in enhancing security and safety.^60–62^ Moreover, ensuring security in high-throughput public areas such as airports demands an efficient system for the swift detection of, and response to, toxic materials and vapors, chemical agents and oxidizers. This need is addressed by RFID-based chemical sensing systems, which enable rapid identification and mitigation of potential threats enhancing overall safety and security measures. The biochemical sensors integrated into these wearables help to monitor the level of fitness of workers, similar to applications in sports, leading to the prevention of injuries and accidents.^63^ Various risk factors, *i.e.* drugs and stress along with physical exertion and hydration level, can be detected non-invasively through saliva, sweat, urine and tears. The emotional state of workers can be non-invasively assessed by measuring the level of specific hormones (dopamine, cortisol and serotonin). This approach contributes to the safety and well-being of workers.^51,64^ Moreover, key environmental pollutants such as CH_4_, SO_2_ and NO_2_ may be detected using compact, wearable gas detection systems, and wearable air quality monitors can gather pollution data from, *e.g.*, urban centers, to yield safe environments for healthy functioning.

## 3. Wearable electrochemical sensors for biomarkers

More recently, advancements that offer non-invasive monitoring of biomarkers find applications in health risk prediction and diagnosis. These sensors monitor biomarkers in biofluids such as saliva and sweat as well as cellular biomarkers. The flexible sensors fabricated with nanomaterials enable on-spot biomarker detection and fast analyte tracing. For example, these wearable sensors monitor nitric oxide (NO) released from endothelial cells, offering insights into vascular functions. The ongoing research aims to improve accuracy, sensitivity and specificity for biomedical applications.

### 3.1. Glucose detection

Glucose is one of the most commonly detected biomarkers for people suffering from diabetes. Monitoring the glucose levels in interstitial fluid helps to manage and control diabetes in real time.^65^ However, other physiological fluids, like urine, breath, and sweat, can also contain biomarkers for glucose and may be explored for concurrent detection of glucose.^66^ Wearable glucose sensors and smart watches based on graphene and gold composites are one of the most popular noninvasive glucose monitoring devices in the market. A bilayer formed from graphene doped with Au and Au mesh results in enriched performance as compared to undoped graphene. This is detected electrochemically using a stretchable device for monitoring glucose in sweat. This stretchable patch is then connected to a smart phone for receiving electrical signals. Besides minimizing temperature, pH, and humidity, the patch comprises polymeric microneedles for the delivery of metformin to control glucose levels. The microneedles are thermally activated.^13,14^ Similarly, a stretchable microfluidic device integrated into a nanoporous Au electrode in a substrate of PDMS is employed for the collection of sweat and monitoring of glucose. For onsite detection, the sensing platform in the form of patch was connected to an electrochemical analyzer *via* Cu wires and then wirelessly monitored *via* smart phone applications.^67^ A sensor was developed to detect glucose in saliva by incorporating it in a mouthguard support, which was initially tested in a phantom jaw. This constitutes a working Pt electrode coated with glucose oxidase enzyme (GOX) and a reference electrode formed on polyethylene terephthalate glycol and seamlessly integrated to a wireless system. The sensor can work in the range of 5–1000 μm L^−1^ of glucose and hence offers a painless method of sensing and monitoring glucose in dental patients.^68^ A non-invasive wireless sensor powered by radio frequency (RF) waves was developed by immobilizing activated GOX for determining the glucose levels in tears seamlessly. This innovative sensor was tested on a polymer-based model eye and also for protein fouling, ageing and temperature fluctuations, demonstrating good repeatability within the range of 0–2 mM.^69^ Remarkably, the sensor was able to read out signals over a distance of several centimeters, highlighting its potential for remote monitoring.

### 3.2. H_2_O_2_ detection

*In vitro* monitoring of cellular biomarkers in cancer cells offers significant benefits by providing insights into the process of signal transmission within the cells. This approach offers valuable insights for both cancer diagnosis and treatment aiding in the understanding of cancer mechanisms and its progression, and hence developing more effective therapeutic strategies. Among biomarkers, H_2_O_2_ serves as a reactive oxygen species (ROS) precursor secreted by liver cells, providing information about oxidative stress and cellular signaling pathways implicated in cancer development and progression. As cancer cells produce more H_2_O_2_ than normal cells as a result of their abnormal growth and proliferation, it continues to accumulate within the cancer cell, potentially leading to the development of serious diseases.^61^ Various flexible wearable electrochemical sensors are fabricated using specific nanomaterials for sensing H_2_O_2_ released from different cells. For example, paper-based electrochemical systems integrated with different nanomaterials are used for on-spot diagnosis.^70,71^ In such systems, flexible microelectrodes are preferred over rigid electrodes to prevent damage or puncture of the cell membrane. For instance, self-supporting graphene paper electrodes,^72^ 3D graphene assemblies such as graphene aerogel, graphene network, and graphene foam^73,74^ as well as fiber-based microelectrodes are commonly utilized.^75^ The fiber-based microelectrodes appear to be particularly compatible with soft tissues for intercellular and near-cellular detection.^76,77^

**Fig. 2:**
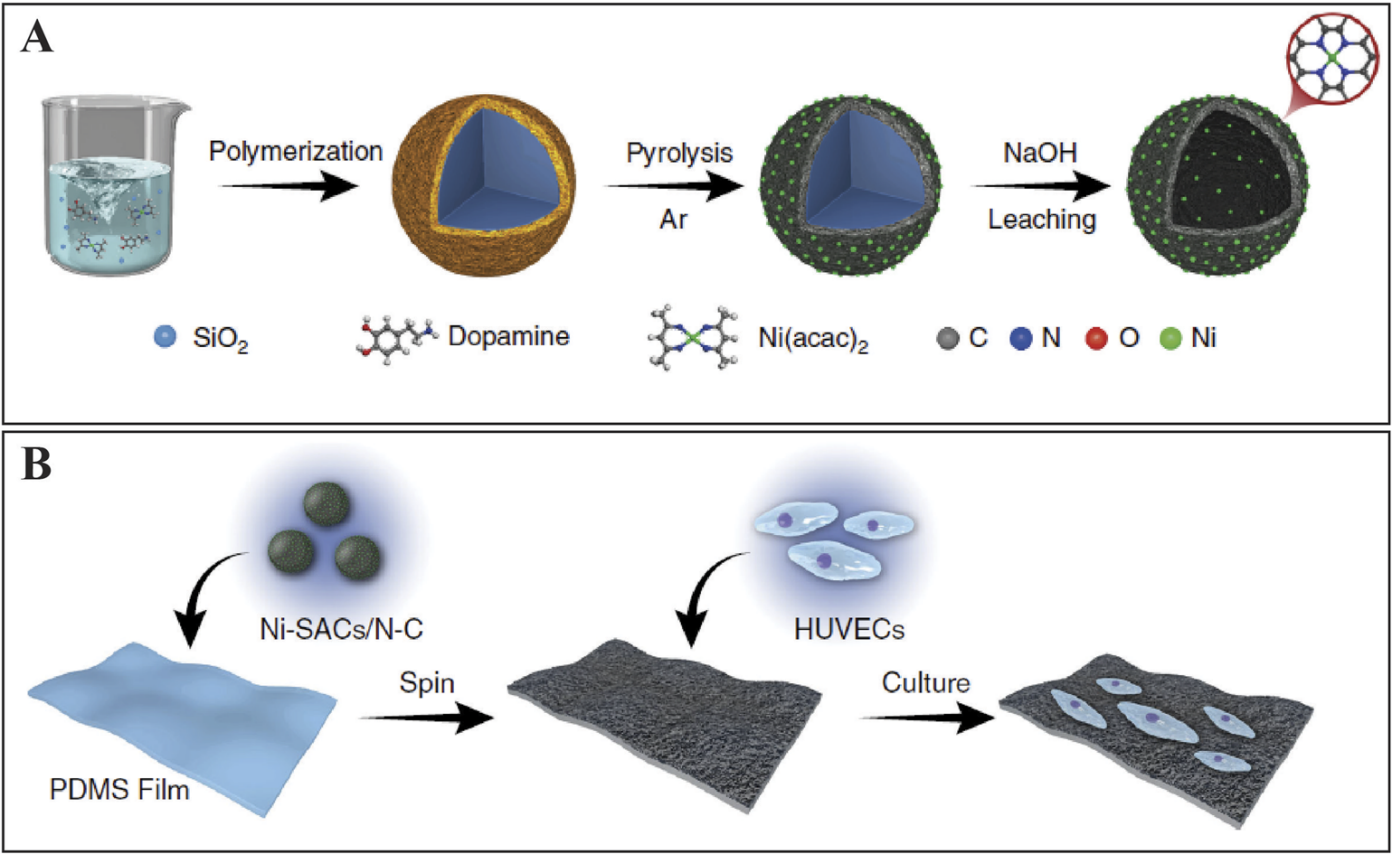
(A) A flexible film electrode with nano-Au/CNTs/PDMS for the onsite analysis of hydrogen peroxide production from HUVECs in the stretched state. (B) Graphical representation of (A) Ni SACs/NC composite synthesis and (B) the sensor to sense NO and HUVECs culture. Reproduced from ref. 80 with permission from Springer Nature Limited, copyright 2020.

Moreover, various fabrication materials are employed to ensure the system's sensitivity. PDMS is engaged as a substrate for stretchable membranes in flexible electronic devices.^78^ Similarly, carbon nanotubes (CNTs) serve as reasonable fabrication materials for flexible microelectrodes owing to their exceptional mechanical strength and large surface area.^79^ As CNTs show relatively low conductivity, conductive metal materials such as Au nanostructures are often electrodeposited onto the CNT films to improve the electrochemical performance for H_2_O_2_ monitoring. A flexible PDMS layered electrode was fabricated following a sequential procedure involving CNTs and Au nanostructures for immediate on-spot determination of H_2_O_2_ from human umbilical vein endothelial cells (HUVECs) and HeLa cells (Fig. 2(A)).^81^ While noble metallic catalysts are expensive catalytic materials, non-noble metallic catalysts can be employed as cost-effective alternatives and serve as an active electrochemical platform for H_2_O_2_ detection in liver cells. For instance, NiCo_2_S_4_@CoS_2_ nanohybrids, synthesized by employing NiCo_2_S_4_ with CoS_2_, exhibit improved redox properties as a result of its high conductivity and mixed valence state.^82^ Currently, the MOFs have been considered suitable fabrication materials because of the enhanced electronic conductance and provision of more active sites for catalysis. Co-based nitrogen-doped CNT materials have also been used for electrochemical H_2_O_2_ detection with consistent linearity values ranging from 0.4 to 7.2 mM. The sensor has exhibited a high sensitivity of 388 mA cm^−2^ mM^−1^, as demonstrated in experiments involving M. D. Anderson-Metastasis breast cancer commonly known as MDA-MB-231 cells and HeLa cells.^83^

Moreover, many researchers have designed numerous nanomaterials for the fabrication of microelectrodes aimed to detect H_2_O_2_ released from living tissues with an ultralow detection limit. Notable examples that have been employed for the same include graphene zero-dimensional (0D) materials and graphene quantum dots (GQDs), exhibiting peroxidase-like activity,^84^ such as peroxidase GQDs with AuPd nanoparticles (NPs).^85^ Similarly, nitrogen-doped CNT composites with AuNPs,^86^ rose flower-like structures (VS_2_@VC@NC flowers) fabricated by PdNPs,^87^ and coral-resembled assemblies of carbon nanospheres incorporating Pt NPs wrapped around hollow carbon tubes,^88^ have also been employed for detection of H_2_O_2_. Furthermore, the combination of MnO_2_ nanowires (NWs) on a graphene fiber (GF) modified with AuNPs (MnO_2_-NWs@Au-NPs@GF)^89^ and an N, B co-doped GF electrode^90^ have been used to distinguish electrochemically between cancer and tumor cells depending upon the difference in the amount of H_2_O_2_ released from these cells. However, it could be said that a substantial body of research is directed toward the expansion of advanced nanomaterials to construct flexible sensors, which serve as a foundation for constructing highly efficient electrochemical sensing platforms for monitoring H_2_O_2_ in the tumor cells and tissues. The work is still ongoing to improve the accuracy and sensitivity of H_2_O_2_ detection in biomedical applications.

### 3.3. NO monitoring

The release of NO molecules from endothelial cells endogenously plays a significant role in regulating vascular homeostasis and immune processes in the body. Its production appears to increase under mechanical forces, *i.e.* shear stress and cyclic stretch, highlighting its role in responding to physiological stimuli and maintaining vascular functions.^91^ However, various effective nanoparticles, *i.e.* metal NWs, NPs, CNTs and graphene, have been used in stretchable electronics as flexible substrates. These substrates fulfill the need for constructing efficient electrochemical sensors capable of detecting mechanically deformed cells.^92,93^ Au NPs applied on the PDMS membrane were employed for NO detection by consuming ecologically sound ultraviolet ray-supported technology. The approach demonstrated a significant linear detection range from 0.010 to 1.295 mM applicable in both non-stretched and stretched states.^94^ Furthermore, owing to improved electrochemical activity and biocompatibility, single-atom catalysts (SACs)^66^ have emerged as new nanomaterials of interest in various applications including electrochemical sensing, medical treatment and enzymatic catalysis.^95,96^ For example, N-doped hollow carbon spheres with dispersed N single atoms were layered on a flexible PDMS surface. HUVECs were then cultured on this surface to monitor NO release under stretching. It showed a high sensitivity of 430.6 nA cm^−2^ mM^−1^ and a substantially low sensitivity limit of 1.8 nM. This work unlocks an innovative pathway to manufacture a flexible sensor based on SACs for *in situ* determination. Following the addition of phorbol-12-myristate-13-acetate (PMA) in phosphate buffer saline (PBS), the release of H_2_O_2_ from HUVECs by comparing these conditions with and without catalase was conducted (refer to Fig. 2(B)).^80^

### 3.4. Metal ion detection

Tugba Ozer and coworkers presented an IOT-based wearable and wireless potassium sensor in sweat. Numerous types of graphite were subsequently modified on Stencil-printed carbon electrodes (SPCEs) to get the best sensor response and then added with carbon black to boost their performance. A small printed circuit board (PCB) readout device was connected with a low-power Wi-Fi for instantaneous data transmission to a cellular device app in order to monitor potassium levels using a software algorithm. The sensor displayed a potassium-selective response (<11 s) with an enhanced sensitivity of 56.1 ± 0.7 mV decade^−1^ and a limit of detection (LOD) of 1 × 10^−5^ M showing linearity in the range of 10^−4^–10^−1^ M. The cost of analysis was also reduced (<$25) as compared to other read-out devices. However, the undertaking indicates a crucial step toward the integration of both detection and data display simultaneously, making potassium sensing convenient in daily life.^97^ Similarly, a noninvasive sweat-based electrochemical sensor was reported, which was integrated with multiplexed flexible skin patches to monitor calcium ions (Ca^2+^), potassium ions (K^+^), and pH with IoT connectivity. This set of sensors revealed selective and repeatable tendency. Its sensitivity came out to be high, *i.e.* 38.33 mV dec^−1^ for Ca^2+^ ions, 64.17 mV dec^−1^ for K^+^ ions and – 46.33 mV dec^−1^ for pH levels. Moreover, the sensor set underwent on-body testing during a 60 min running exercise. The results indicated decreased Ca^2+^ ion levels and increased concentrations of K^+^ ions. The pH in real sweat was consistent with expectations. This newly developed sensor holds promise as an essential tool for remotely testing ionic components and studying pH levels in sweat.^98^

### 3.5. Aldehyde detection

The outbreak of pandemic diseases such as coronavirus disease (COVID-19) has underscored the urgent need for reviving our conventional healthcare analysis system, with a focus on reducing the production of waste and subsequent environmental pollution. This can be achieved with the help of electronic technology and modern computing tools such as IoT platforms, large data analysis, very large scale integration (VLSI), machine learning, deep learning, and artificial intelligence or AI to develop a real-life health analysis system.^99^ A cloud-based IoWT sensing system was developed to measure exposure to aldehydes (known airway irritants) among asthma patient's in real-life scenarios. This system employed a wrist-watch like sensor capable of monitoring the concentration of formaldehyde (30 ppb to 10 ppm) in air uninterruptedly, without recharging for 7 days using fuel cell technology. Additionally, the sensor was designed to operate seamlessly for an entire 7 days period, providing uninterrupted monitoring. This sensor was able to upload data wirelessly to a cellphone technology with an android operating system through Bluetooth Low Energy (BLE). This smartphone technology acted as a portal to a cloud-based informatics system for managing the sensor's analytics, data storage and its management. Moreover, the smartphone acted as a doorway to cloud-based IOWT system. This IoT sensing system could further be used to study the epidemiology of asthma, refine asthma management strategies and conduct environmental analysis.^100^

## 4. Monitoring of analytes in biofluids

It is worth mentioning here that biofluids such as saliva, sweat, and tears provide a wealth of information about an individual's health and hence can provide valuable insights into his well-being. Monitoring analytes using electrochemical sensors, coupled with IoT connectivity has emerged as a powerful approach. These biofluids contain essential information about physiological processes, disease markers and metabolic changes. Thus, the synergy of electrochemical sensing and IoT transforms biofluids into dynamic diagnostic tools, enhancing personalized healthcare and disease management.

### 4.1. Sweat

The growing occurrence of wearable devices has lately gained interest in immediate health monitoring and tracking body's physiological parameters. A recent investigation has been completed to develop a cost-effective device for sweat analysis (glucose and potassium ions) using selective electrochemical methods and microfluidic techniques. The wearable device monitors the levels of potassium and glucose ions in sweat when the body is engaged in any physical activity. It then issues a warming signal when the ion level reaches an experimentally set threshold concentration of 7.5 mM for potassium ions and 60 or 120 μM for glucose. The signal notifies the users of possible hypoglycemia and dehydration. Such advancements in wearable technology are potentially significant for customized health management and safeguarding, upgrading overall welfare, and adjusting performance in physical performances. Recently, enzyme-free glucose detection in sweat was reported by employing a flexible integrated wristband-based wearable sensor. This sensor was also able to transfer information to an app through Bluetooth for management regarding remote health. Moreover, during glucose detection, the evolution of hydrogen bubbles restricts the reproducibility of the electrode, which was solved by using Co MOFs encapsulating Pd NPs (Pd@ZIF-67-Zinc Imidazolite framework) as electrocatalysts. This was integrated into a sweatband along with a pliant printed circuit board for the onsite analysis of glucose in sweat.^101^ The device was reported to sense glucose under physiological pH conditions unlike stated in reports before, by nonenzymatic glucose sensors that operate in alkaline buffers. Additionally, it enabled wearable, maintenance-free tracking of glucose in perspiration over an extended period and holds promise for non-invasive clinical analysis and application in sports.

### 4.2. Saliva

Saliva is one of the non-invasive fluids with potentially significant biomarkers for unveiling physiological conditions. It majorly consists of proteins, aqueous, organic and inorganic elements. Saliva is used as a biofluid substitute for assessing disease biomarkers by employing simple, non-invasive testing without instigating pain. The changes in the concentration of saliva can be used to diagnose diseases and for health check in detailed analysis since blood constituents can pass into saliva *via* facilitated diffusion.^102^ To date, it is challenging to monitor electrolytes in saliva using a sensor. However, researchers are employing electrochemical sensors in devices for this purpose. A study reported fully integrated wearable sensor arrays for real-time measurements of targeted analytes in saliva. Further, the COVID-19 pandemic episode highlighted the urgency to revamp our healthcare systems where a lot of environmental toxic wastes are generated, which results in infection outbreaks among patients who have to visit a healthcare official. Hence, to address these issues, nano-biosensors have been developed for robust and sensitive detection of diseases. These biosensors are in the form of wearables that enable remote health monitoring by using IoT and machine learning, and hence, help in real-time diagnostics.^99,103^ A study reported fully integrated wearable sensor array for the real-time measurement of targeted analytes in saliva. The fully integrated sensor for remote, non-intrusive, and concurrent measurement of several metabolites and electrolytic ions such as Na^+^ and H^+^ in saliva or sweat was developed by combining sensing, electronic and microfluidic units in a continuous mode. The efficacy of the developed sensor was assessed for a multitude of electrolytes in healthy individuals and gout active patients by administering a purine-rich meal and medicated-care control regime, respectively. Such monitoring of numerous electrolytes and metabolites through single universal integrated on-body sensors might lead to better comprehension of the biomarkers in a human body with an added benefit of personal health management^103^ In a recent study, an electrochemical sensor based on graphitic carbon nitride was developed to detect interleukin-8 in saliva. Interleukin-8 is a cytokine linked to oral cancer. The sensor is highly sensitive with an excellent LOD and is a great advancement towards point-of-care diagnostics.^104^ In another study, a stationery electrochemical power-angle paper-based sensor was reported to test iodide and dopamine concentrations in saliva samples. Following low-cost miniaturization, the hydrophobic layouts on paper substrates were simple and robustly drawn with a crayon pencil devoid of cutting-edge tools.^102^

### 4.3. Tears

Non-invasive detection of biofluids is possible by various sensing platforms, while analyte monitoring for different physiological biomarkers can be carried out on different body sites.^100^ The tear is an interstitial fluid comprised of metabolites, electrolytes, and proteins obtained from glands such as goblet cells, lacrimal glands and ocular surface epithelial cells.^105,106^ Although there are generic differences between the composition of blood and human tears, they correlate due to leakage of certain metabolites into tears while blood is supplied to the brain. This makes it easier to create tear proxies to simulate metabolites' blood level. A study has reported the use of either functionalized contact lenses or merging them with miniscule sensors (such as glaucoma detection) for diagnostic monitoring of tear metabolites.^107^ Recent developments in biosensors, transparent electrode materials and telecom technologies have led to intelligent contact lenses that are controlled remotely and able to provide continuous tear-glucose readings (research done by Google and Novartis). Yao *et al.*,^108^ have also reported polyethylene terephthalate (PET) contact lenses for glucose monitoring using a simple three-electrode electrochemical sensor.^109^ Photoresist and thermal evaporation of titanium and platinum on a PET-coated wafer was used to make the electrodes. The glucose oxidase was immobilized by covering the contact lens surface with a passivation layer of titanium oxide. Probable interferences from ascorbic acid (AA) and lactate were reduced using a Nafion layer.^110^ Wearable electrochemical sensors potentially measure biomarkers (cortisol, glucose, *etc.*) in individuals assisting health information management and fitness. These function by operating at low power by applying low potentials, and hence, they are safe for various applications. Basically, electrochemical sensors employ electrodes to convert a chemical reaction into an electrical signal. In order to enhance electrode performance, transducers are immobilized with bioreceptors (enzymes, antibodies, aptamers, *etc.*) such that their activity is not hindered. Consequently, these transducer materials are required to have a high electrical conductivity that could detect small electrical changes at the electrode–electrolyte interface. Moreover, these can be miniaturized with ease, are economic and robust, and exhibit compatibility with wearable electronics that makes them ideal for the body.

## 5. Novel interfaces and advanced materials

The signal transmission and transduction in biosensing systems are significant for stable, accurate and sensitive outgoing signals. The electrical conductivity of a material is considered a basis for ensuring standard signal transmission. Moreover, the flexibility and adhesiveness of materials need to be investigated, as wearable devices are supposed to fix tightly to human skin to prevent signal interference and distortion during transmission. The hydrophobicity of the materials acts as a support in preventing interferences due to sweat. Consequently, fabricating engineered materials possessing any of the above-mentioned desired qualities is necessary for wearable sensing systems. In this regard, researchers have been getting inspiration from nature as numerous organisms occurring in nature have evolved as ingenious, complex and robust sensing systems. For instance, the lateral line system of fishes can sense changes such as velocity and viscosity of nearby running water, assisting in finding direction and detecting objects in water.^111^ Consequently, the enhancement of integration capability and stability is significant for the advancement of the sensor system with regard to advanced materials for wearable electrochemical sensors, as listed in Table 1 in Section 5.5.

**Table 1:** Wearable electrochemical sensors for tailored health monitoring

Sr. No.	Analyte	Modification	Detection technique	LOD	Ref.
1	Tyrosinase	CP/catechol-agar	Chronoamperometry (CA)	Not listed	112
2	Lactate	CNTs/Ti_3_C_2_T_*x*_/PB/CFMs/chitosan-LDH-BSA	CA	Not listed	113
		GO NSs/PA/PANHS/LOx	EIS	1 Mm	114
3	Glucose	CNTs/Ti_3_C_2_T_*x*_/PB/CFMs/chitosan-GOx-BSA	CA	Not listed	113
		CNFs/chitosan-GO/GOx	Colorimetry	0.1 mM	115
		PET/CoWO_4_/CNTs/PANI/Silbione	CA	1.3 μM	116
		PB-CI/BSA-AO_*x*_/PU/PVA	CA	Not listed	117
		Multiplayer PB/GOx	CA	1 μM	118
		GO_*x*_-AuNPs/N-GQDs-PMOF	Amperometric	0.7 μM	119
		GOD-GA-Ni/Cu-MOFsFET	Amperometric	0.51 μM	120
4	Radial muscle contraction	PLA/paper-Ti_3_C_2_T_*x*_/PLA	CA, frequency	10.2 Pa	121
5	Urea	CNFs/chitosan-GO/urease	Colorimetry	30 mM	115
6	Potassium	CI/PDMS/LSG/MWCNTs	Potentiometry	10^−4.9^	122
7	Cortisol	PDMS/Ecoflex/AgNPs/MIP/NIP	Conductivity	2 ng mL^−1^	123
8	Ph	PET/CoWO_4_/CNTs/PANI/Silbione	Potentiometry	Not listed	116
9	Alcohol	PB-CI/chitosan-Gox/agarose	CA	Not listed	117
10	Hydrogen peroxide	PMMA/PB-CI/Whatman/PMMA	CV, CA	Not listed	124
11	SARS-CoV_2_ virus	Apt/polyUiO-66 @AgNPs	DPV	Not listed	125
12	H_1_N_1_ virus	Ab/poly UiO-66 @AgNPs	DPV	49.4 fg mL^−1^	126
13	Tuberculosis antigen	Aptamer/HRP/AuNP/UiO-66-NH_2_	DPV	10.0 fg mL^−1^	127
14	mecA and nuc gene	UiO-66/BMZIF-derived NPCs	DPV	3.7 fM	128
15	*Escherichia coli* K12	MOFs-Ab-*E. coli*-AuNP	CV	1.0 cfu mL^−1^	129
		Ab/Cu_3_(BTC)_2_-PANI	Impedimetric	2.0 cfu mL^−1^	130
16	*Vibrio parahaemolyticus*	Fe_3_O_4_@NMOF-Apt	SWV	3.0 cfu mL^−1^	131
17	*Pseudomonas aeruginosa*	Cu-ZrMOF@Aptamer@DNA	DPV	Not listed	132
18	Carcinoembryonic antigen	Pt@CuMOFs-hGq-GOx	Impedimetric	0.023 pg mL^−1^	133
19	T4 polynucleotide kinase	Fe-MOF/AuNPs/DNA	DPV	3.5 × 10^−4^ U mL^−1^	133
		FeMOF@AuNPs-hairpin	CV	2.344 × 10^−4^	134
20	Mucin	Cu-MOF-GO	DPV	0.033 pM	135
21	Thrombin	AP II/AuNPs/Ni-MOF	DPV	0.016 pM	136
22	Uric acid	Au@NC@GC	DPV	10.0–150.0 μM	137
23	Dopamine	ZIF-8/PPy@SiO_2_-MEPCM and laccase	DPV	0.0069 μM	138
		Mn-BDC@MWCNTs	DPV	0.002 μM	139
24	Lactose	Co-hemin MOF/chitosan/PcCDH	Amperometric	4.0 mM	140
25	Carbohydrate antigen 125	MOF-808/CNT/Ab	DPV	0.5 pg mL^−1^	141
26	Ciprofloxacin	NH_2_–UiO-66/RGO	ASV	6.67 nM	142

### 5.1. Metal–organic frameworks (MOFs)

**Fig. 3:**
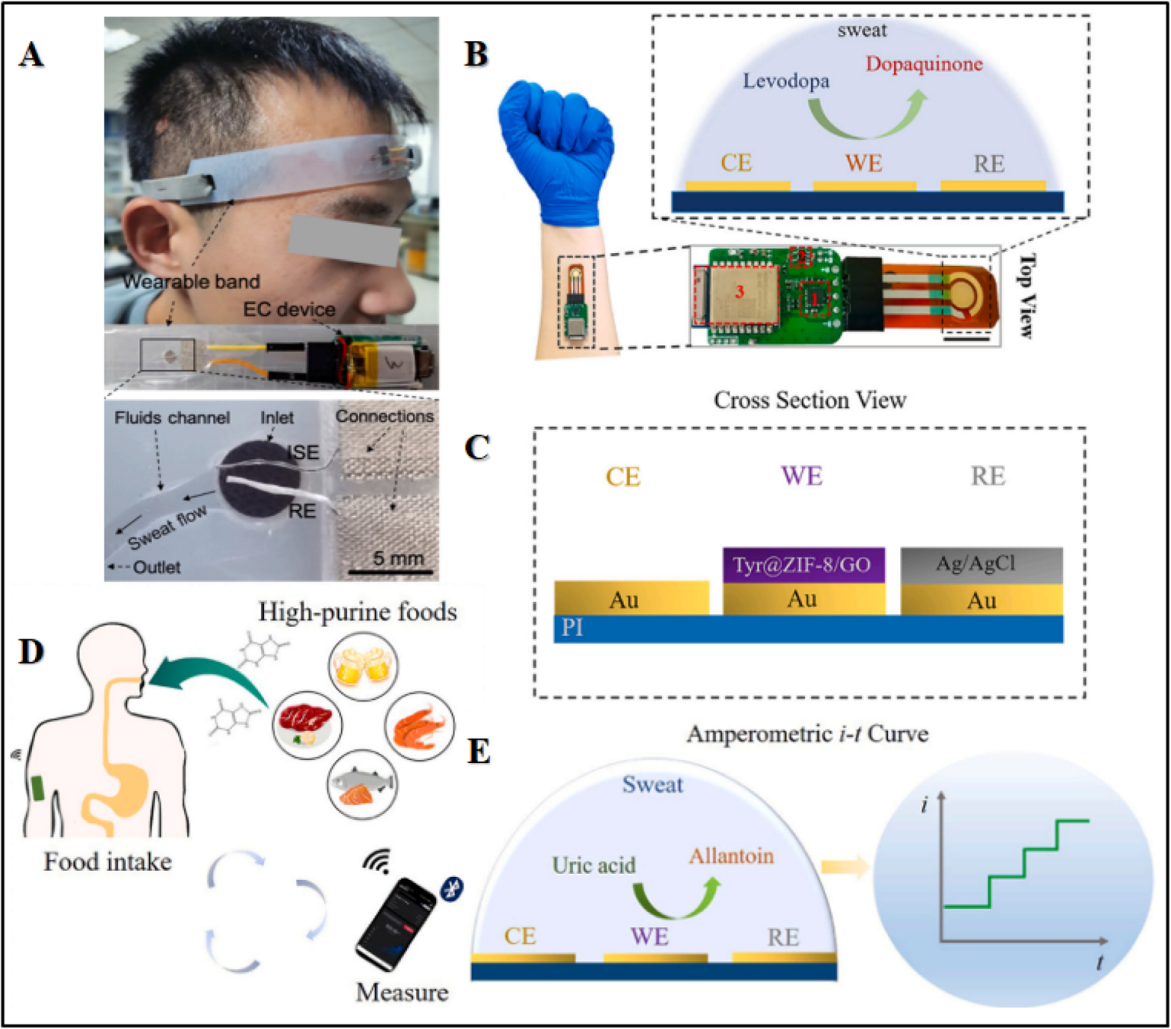
Illustration of (A) a wearable band-based sensor to detect on-body sweat. Reproduced from ref. 106 with permission from Elsevier, copyright 2022. Wearable levodopa sweat sensors with construction details. (B) Levodopa sensor. (C) Functionalized electrodes. Reproduced from ref. 143 with permission from the American Chemical Society, copyright 2019. Mechanism of uric acid sensing. (D) Real-time uric acid detection in sweat with high-purine food intake. (E) Sensing process of the uric acid sensor. Reproduced from ref. 144 with permission from Elsevier, copyright 2022.

Despite their low electrical conductivity and fragile mechanical characteristics, MOFs are widely used for chemical sensing owing to their selectivity and precision for the detection of analytes, for their outstanding catalytic capabilities and energy generation. For instance, Shuqi Wang and coworkers developed fiber-like Nickel triphenylene-fused metal catecholate (NiCAT4) on CNT fiber-ion selective electrode (CNT@ISE)-NiCAT4@CNTF-RE and Nafion/NiCAT4@CNTF-ISE, for fabricating a sensor band intended to be worn on the head in order to monitor continuous flow of sweat without direct interaction of the skin, as shown in Fig. 3(A).^106^ This device showed biocompatibility, good flexibility, and mechanical stability. A small electrochemical workstation attached with this device allowed it to connect the on-spot potentiometric signals to the smart phone *via* wireless connection. In this way, the wearable sensor monitored the sweat with smooth and spikes-free signal responses. The calculated sodium concentration ranges up to 80 mmol L^−1^ in sweat, which is in line with the normal Na^+^ level (10–90 mmol L^−1^) during active sweating session. To assess the stability of the as-fabricated material, constant-current chronopotentiometry was employed to study Nafion/NiCAT_4_@CNTF-ISE and Nafion@CNTF-ISE. Furthermore, improved sensing performance was observed, attributed to enhanced double-layer capacitance and improved conductivity of solid-contact transducers based on NiCAT_4_ and Nafion. The negative potential shift was observed for both NiCAT@CNTF-ISE and CNTF-ISE in the interfering ion solution as compared with the primary ion solution, indicating the presence of hydration spheres at the electrode and solution interface.^106^

Similarly, Jingyu Xiao and coworkers displayed a wearable sweat levodopa (LD) sensor. The LD is the first-line drug for the treatment of Parkinson's disease. The sensor analyzes sweat and contains a flexible electrode to analyze LD that is integrated into a minute electronic circuit for its wireless analysis (Fig. 3B). The WE was fabricated by immobilizing several layers of GO/MOF/enzyme on the surface of the electrode, in the form of a composite using screen printing technology to ensure the accuracy and selectivity for LD in sweat. The functional surface of the electrode was modified with tyrosinase coated on ZIF-8/GO. The role of ZIF-8/GO composite was to provide a wide interface for the incorporation of tyrosinase in order to enhance the sensing performance, while tyrosinase was used to catalyze the oxidation of LD to dopaquinone. This monitoring of LD was carried out in real time by studying the current response of this reaction.^107,143^ Moreover, Shu and coworkers developed a rapid, uninterrupted, and highly accurate method of detecting glucose concentrations in sweat by fabricating a highly flexible fiber-based wearable sensing device using Ni–Co MOF/Ag/rGO/PU. The Ni–Co MOF's fiber-based sensor displayed a high surface area and outstanding catalytic activity to achieve a linearity of 10 μM–0.66 mM and a sensitivity of 425.9 μA mM^−1^ cm^−2^. The sensor was highly selective with excellent storage stability along with mechanical deformation strength, including remarkable bending and stretching stabilities.^145^ Additionally, Zhao *et al.*, were the first to develop a hybrid core–shell multifunctional MOF-based 3D CNF network (CNFN) by specifically using copper catecholate (Cu-CAT) employing hydrothermal and electrospinning techniques.^109^ The flexible CuCAT@CNFN-based wearable pressure sensor demonstrated a fast recovery time of 0.24 s, high sensing range from 0.5 kPa to 60 kPa, exceptional stability for over 5000 cycles and reliable air permeability, and can effectively captivate joint movement such as finger gestures. In future, this technology could be applied in smart home gadgets by integrating pressure sensors, supercapacitors (SCs) and solar cells into multipurpose, self-powered systems.

Wearable and flexible real-time uric acid (UA) sensors in sweat were developed by integrating the sensing platform uricase@MAF-7 (3-methyl-1,2,4-triazolate framework (MAF)) with a microchip and wireless board for the collection of sweat and monitoring particularly after consuming high-purine meals, as shown in Fig. 3(D). The UA is a byproduct of metabolism of purine and an increase may indicate consumption of purine-based diets. In this study, MAF-7 was integrated with an urease enzyme, where the composite MOF/GO(graphene oxide)/enzyme was then deposited onto a gold working electrode (WE) to realize a higher sensitivity and sustain enzyme functioning in severe environments.^144^ The WE was fabricated with several layers of the composite, as shown in Fig. 3(C). The inclusion of MAF-7 improves the sensor's antifouling performance and stability by protecting and loading the enzyme onto the sensor. Further modification with a Nafion layer contributed to these improvements.^110,146^Fig. 3(E) depicts the oxidation of UA to allantoin by the respective catalyst uricase. The inaccuracies due to the evaporation of sweat and contamination of skin in conventional sweat sampling were reduced by microfluidics-based sweat sampling.^111,147^ Embedded on a microfluidic chip was a microchamber and several microchannels (the inlet port) required to collect samples of sweat. Capillary action facilitated the transport of sweat to the microchamber through the microchannels, while excess sweat was drained *via* the outlet. Furthermore, the feasibility of this microfluidic chip was assessed by introducing a blue colored water drop at the input and checking whether it fills the chamber or not. No visible leakage through the whole chamber ensured reliable sweat sampling. The collected sweat samples were transferred wirelessly to the user's computer or mobile device through Bluetooth.^148,149^ Similarly, Zhu and coworkers used a Pd core-shelled NP electrocatalyst in Co ZIF-67 for the analysis of wearable glucose perspiration by nonenzymatic, water splitting-based electrochemical sensors.^101^

### 5.2. Carbon nanotubes (CNTs)

Various nanomaterials including NPs, CNTs, molecularly imprinted nanostructures, quantum dots, inorganic nanorods/nanowires, and many more have been used widely as fabrication materials to enhance the sensitivity of interface of the sensor for biomedical diagnostics.^150–155^ Recently, electroactive nanoprobes have been reviewed broadly for the determination of analytes of interest by electrochemical sensing.^156–159^ They also provide new tools for the diagnosis of hepatocellular carcinoma,^160^ analysis of living cells integrated with electrochemical cytosensors,^161^ and detection of cancer cells based on folate receptors.^162^ Such electrodes have also been used for the analysis of cancer biomarkers and anticancer drugs, showing their great analytical performance.^163^ Moreover, Pingarron *et al.*, have succeeded in expanding the portfolio of biosensing devices to provide precise information about the specific location of tissues. In this context, nanomaterials have been recognized as outstanding materials for effective drug delivery by preventing biosystems from the non-specific interactions and reducing multi-drug resistance. Additionally, nanomaterials enable direct drug delivery to the ailing tissue in the organism, enhancing therapeutic efficacy.^164,165^ In addition, thin films of single-walled carbon nanotubes (SWCNTs) were used as stretchable materials in wearable electrochemical health devices. A dry and clean fabrication process was selected for the formation of pristine and high-quality SWCNT films incorporating a protective layer to mitigate the risk of surface contamination from other fabrication processes during electrochemical activity.^166^ Such electrodes were found to exhibit a low electron transfer rate. In a further study, the electrochemical response of SWCNT electrodes was improved by the successful functionalization of electrodes with a H_2_SO_4_ solution using inner redox probes such as Fe(CN)_6_^4−/3−^. This electrochemical functionalization of the working electrode came out to be effective for improving the limit of detection to about ∼100 nM for the detection of dopamine with such a flexible SWNT electrode.^167^

### 5.3. Polymer or polymeric membrane

The demand for innovative and highly efficient modifiers with excellent redox, thermal and chemical resistance for the electrode is often met by using polymers such as thin films of modern stimulus-responsive polymers,^168^ synthetic polymers^169^ and polymer nanocomposites.^170^ Among them, photoactive polymers as well as molecularly imprinted polymers are well designed and environmental friendly polymers having the potential to be used for many selected applications.^171^ One application of polymer membrane layers used as a sensing platform on the electrode surface is to develop a reliable, portable and low-cost diagnostic platform for biomarkers in human fluids.^172^ All of these polymer membranes basically act as a protective barrier against interferences caused by human fluids such as serum, blood, urine or plasma samples on the electrode surface, allowing for the monitoring of analytes.^173^ Additionally, when serving as an external protective layer for electrodes, they ensure accurate measurements of signals originating from the desired molecule by shielding the surface of electrochemical biosensors against unwanted non-specific interactions, fouling, and signal interferences.^174^ Electropolymerization technique is extensively used for the electrode surface assembly owing to its wide range of advantages including easy implementation, signal enhancement, high reproducibility, compatibility with transducers and protection of bio-recognition elements. Electropolymerized materials on DNA receptor elements have been incorporated successfully to form innovative polyelectrolyte complexes.^65^ Various polymers including polypyrrole, polyaniline, polyphenazines, polythiophene and their derivatives have been used as sensing materials. Moreover, Au NPs and poly-aminothiophenol^175^ as well as polymeric and oligomeric forms of lactic acid^176^ have been electropolymerized for the formation of a biocompatible, cost-effective and durable film on the electrode surface. Another research group introduced a wearable electrochemical sensor based on polyaniline thin films immobilized on the flexible polyethylene terephthalate substrate coated with indium tin oxide (ITO) for sweat analysis. The potentiostatic deposition technique was used to obtain polyaniline thin films by applying a constant potential of +2 V *vs.* SCE (saturated calomel electrode) initially for 90 s, followed by its modification with reduced graphene oxide at +8 V of constant potential *vs.* SCE for 200 s. Subsequently, the electrodeposition of polyaniline thin films was conducted to improve the sensor performance. This fabricated electrode showed good response with 62.3 mV pH^−1^ sensitivity (approximately similar to Nernstian response), improved selectivity among other ions with minimal interference effect and 3.8% reproducibility.^175^

### 5.4. Transition metal chalcogenides (TMCs)

Two-dimensional materials are considered promising for the fabrication of stretchable wearable sensors for safety and health monitoring. Conventionally, the materials used are polymers, silicon, and inorganic oxides, but unfortunately such materials have encountered problems such as low conductivity, high cost and rigidity that impede their practical use. The TMCs belong to such a class of 2-D compounds (metal sulfides) that have demonstrated promising results in integrated wearable sensors owing to their unique layered structure. The TMC exhibit adjustable properties such as transparency, band transitions and elasticity, which make them promising materials for wearable sensors.^177^ Although it is still challenging to manufacture high-quality wearable sensors due to toxic solvents and crystal formation of TMCs, a study reported zwitterion cocamidopropyl betaine (CAB) acting as a surfactant to support liquid-phase exfoliation and homogeneous dispersion of single-phase crystalline TMC in solvents such as water and isopropanol. In the absence of binders or additives, several stable TMC inks were directly produced for deposition on flexible substrates for wearable sensing electronics.^175,178^

### 5.5. MXenes

Continuous and real-time sensors have been receiving much attention lately, in biomarker monitoring, therapeutic agent tracking and toxicity assessment. Nevertheless, such sensors are still in development stage regarding on-site applications due to several factors including short service life liability to fouling, poor repeatability, signal drifting, *etc.* Furthermore, present successful methods suggest more sample pretreatment and timely procurement of testing results. In this regard, a class of novel 2D nanomaterials such as transition metal carbides, nitrides and carbonitrides referred to as MXenes exhibit high electrical conductivity and a large surface area due to abundant terminal groups. These characteristics enable them to serve in various functions and structural designs, rendering them as excellent candidates for the construction of wearable sensing devices. The electrical properties of MXenes can be tailored to selectively adsorb biomolecules (*e.g.*, glucose and dopamine) *via* surface modification. MXenes have been engaged to fabricate biosensors for various biofluids. For example, an organ-like titanium carbide (Ti_3_C_2_T_*x*_) is used as an electrochemical sensing matrix for electrocatalysis and enzyme loading. The first use of enzyme-based detection mechanism of H_2_O_2_*via* Ti_3_C_2_ was reported by Yang and Gao,^3^ wherein hemoglobin was immobilized on the MXene surface facilitating direct electron transfer and target molecule detection. Hemoglobin immobilization for the detection of nitrite was also reported, wherein Ti_3_C_2_T_*x*_ was used to fabricate a mediator-free biosensor. Similarly, other enzymes such as acetylcholinesterase (AChE) and tyrosinase were successfully immobilized on MXene surfaces.^170,173^ Chemical additives have been shown to increase the sensitivity and detection performance of MXenes. For instance, TiO_2_ nanoparticle-modified Ti_3_C_2_ generated highly improved detection of H_2_O_2_. Gold-based MXene nanocomposites (Au/MXene) have been reported for biosensing applications *via* glucose immobilization. The Au/MXene-based platform was shown to possess excellent electrocatalytic properties, and enhanced electron transfer between the GOx enzyme and the WE was observed. Surface treatment such as HF etching has been shown to enhance the electrochemical properties of Ti_3_C_2_T_*x*_ for the construction of an electrochemical biosensor to detect carbendazim (CBZ).^179,180^ Similarly, Nafion-coated Ti_3_C_2_T_*x*_ was employed to construct a high-performance electrochemical detector for the neurotransmitter dopamine, demonstrating an LOD of 3 nM. Nobel metal nanoparticle (Au, Pt and Pb)-based structures were constructed on MXene sheets to yield a superoxide detecting biosensor that exhibits excellent linearity in the range of 0.4–9.5 × 10^−6^ M and an LOD of 0.2 × 10^−6^ M with enhanced catalytic interactions during the sensing mechanism. A novel electrochemical biosensor with urease-immobilized MXene nanosheets integrated in a microfluidic chip was developed for analysis of whole blood.^19,181^ Sensing platforms based on the nanocomposites of MXenes provide improved detection performance, high repeatability and stability, and are constructed by combining Ti_3_C_2_T_*x*_ with various nanomaterials. As an example, a nanocomposite consisting of platinum, polyaniline and Ti_3_C_2_ (Pt/PANI/MXene) was engaged for the detection of H_2_O_2_ and lactate. The modified setup provided improved response towards H_2_O_2_ with a linear range of 0.0005 to 5.0 mM for lactate. Another hybrid nanocomposite sensor, composed of palladium NPs on MXene sheets, demonstrated electrochemical detection of l-cysteine, with the sensing range of 0.5–10 μM and an LOD of 0.14 μM.^182^ A study of electrochemical detection of the neurotransmitter epinephrine was conducted using graphene oxide (GOx)/Ti_3_C_2_T_*x*_ MXene (GMA) sheets situated onto indium tin oxide (ITO) to construct a GMA/ITO electrode. A similar study using an MXene/Graphene hybrid was reported for the detection of glucose from GOx, exhibiting enhanced electrocatalytic capability for glucose sensing. In another study, the non-enzymatic detection of glucose was achieved using a Ti_3_C_2_T_*x*_ MXene/Ni–Co–double layered hydroxide (LDH) sensor demonstrating excellent linearity in the range of 0.002–4.096 mM. It was further proven that the presence of functional groups on the surface of MXenes enhances their ability to immobilize active analytes.^183^ Another MXene hybrid combining Ti_3_C_2_T_*x*_ nanosheets with immobilized tetrahedral DNA structures demonstrated mycotoxin sensing using a gliotoxin aptamer with a sensing range of 5 to 10 pM with an LOD of 5 pM. A high-performance hybrid sensing platform for the detection of acetaminophen was reported where *in situ* crosslinking of amino-functionalized MWCNTs with polydopamine-functionalized Ti_3_C_2_T_*x*_ showed increased mechanical stability and excellent electrochemical response. A similar combination of electrocatalytic MXene/NH_2_-CNT sensor for selective sensing of fisetin was reported, showcasing enhanced surface area. Likewise for monitoring cholesterol, a nanocomposite-based biosensor was developed by using Chit/Ti_3_C_2_T_*x*_ to immobilize cholesterol oxidase (ChOx) enzyme. The study showed enhanced electrical conductivity, favorable selectivity and practicability. Moreover, Au/MWCNTs/Ti_3_C_2_T_*x*_ was reported for the detection of HM ions such as Cu and Zn. Furthermore, mixed-phase MXene nanosheets were reported for the simultaneous detection of multiple biomolecules such as AA, dopamine and UA.^184^

Despite the various reported works exhibiting the development of MXene-based electrochemical biosensors, there is only a single case of wearable biosensor based on MXenes that we could unearth. In the study, a wearable, multifunctional biosensor was presented, designing a unique composite of MXene/Prussian blue (Ti_3_C_2_T_*x*_/PB)^1^ to diagnose glucose and lactate in sweat, in order to overcome issues in sweat-based sensors such as compromised sensitivity and short detection range. The reported novel modular design, showcasing a three-phase CNTs/Ti_3_C_2_/PB electrode, exhibited enhanced sensor performance and stability. The sensor was shown to simultaneously measure signals of glucose and lactate with high sensitivity. The overall sensor design consisted of a sweat-collecting layer, sensing layer and covering layer. Therein, sweat collection is followed by pH, glucose, and lactate level detection *via* the insertion of active sensors. The process is facilitated by soft silicon rubber allowing oxygen diffusion towards the active sensor layer for protection from an open environment. Electrochemical nanobiosensors with MXene additives have shown promise to detect different types of biomolecules such as piroxicam, AA, and UA.^185^ In another investigation, nanocomposites based on MXene/CNT/PB were designed to fabricate a nanobiosensor for glucose and lactate monitoring.^186^ Primarily, the sensor was modified with H_2_O_2_ secreting enzymes (lactate and glucose) that also cause the ionization of PB dye. The produced ions react with MXenes, initiating a redox reaction with enhanced electrochemical sensing, demonstrating an LOD of 0.33 × 10^−6^ M for glucose and an LOD of 0.67 × 10^−6^ M for lactate.

An MXene- and CNT-based CuMOF composite nanosensor had been fabricated to detect tyrosine. Entrenching MXenes with CNTs prevents their aggregation and altering them with CuMOF porous octahedral particles produces richly porous and catalytic composites. Sensors displayed high stability and linearity in the range of 0.53–232.46 μM with an LOD of 0.19 μM.^187^

An exceedingly responsive electrochemical sensor has been fabricated to detect nitrile *via* a direct green synthesis procedure. Xylan-based CQDs have been used as *in situ* reducing agents for the preparation of CQD-covered gold nanoparticles, *i.e.* Au@CQDs. An electrically conductive MXene had been used as an immobilized matrix for the preparation of an electrode nanocomposite sensor as Au@CQDs/MXene, which was later loaded onto GCE. The fabricated sensor had been amply responsive, showed extensive ranged linearity, and exceptional selectivity because of AuNP and CQD catalytic activity. Under the most favorable conditions, the linearity for the sensor ranged from 1 μM to 3200 μM with an LOD of 0.078 μM. These values have been superior to most sensors that were reported in the literature by a differential pulse voltammetry (DPV) method.^188^

Table 1 presents a summary of wearable sensors with significant implications for healthcare.

## 6. Advancement in flexible wearable electrochemical sensing devices

As flexible wearable electrochemical biosensors are quite minute and manageable, they can be directly mounted onto the skin for constant noninvasive analysis of analytes in the humans. Their assembly with integrated circuits has displayed robust electronic responses and effective capabilities of data processing. They also provide an important platform to overcome various problems in real-time monitoring and remote health management, combined with precision medicine and flexible bioelectronics technology.^19,181^ Moreover, they have been identified as tools to meet complex medical monitoring requirements due to their improved structure and the progress of flexible electronics and nanomaterials. These flexible biosensors not only represent better sensing ability but also display strong practicability, having wide-ranging applications in clinical and commercial health prospects.^182,189^

The conventional method of sweat analysis has several problems such as cumbersome collection, transportation processes and delayed information. Multiple fabrication techniques have been proved practical for wearable electrochemical sensors to overcome these limitations and offer ways for *in situ* real-time sweat determination. For instance, the progress of micro-devices has been promoted by microfluidics for developing wearable devices that can gather, sample and analyze sweat.^102,176^ Furthermore, the application of near-field communication (NFC) technology offers battery-free alternatives in comparison to Bluetooth technology for developing wireless electronic devices for sensing purposes.^183^ In addition, a steroid hormone was detected by DPV in sweat by employing a flexible cortisol patch device integrated with a NFC chip. It was connected to the surface of skin for data collection that was transported wirelessly to the NFC-based smartphone. However, it provides a solution for analysis of cortisol biomarkers in sweat.^184^ Similarly, the analysis of glucose levels in sweat is widely reported *via* wearable biosensors connected with bioelectronics,^185,190,191^ but the data for nonenzymatic wearable sensors based on bioelectronics and nanomaterials for glucose are rarely reported.

The growth of IoT has made edge computing (involves connecting numerous computing and storage devices to generate a large amount of data) highly significant.^187^ It can be executed by using multiple technologies, *i.e.* fog computing, cloudlets and micro data centers, making networking, storage and computation abilities to be installed at the place of data generating unit.^188,192^ In a healthcare study, a decision-making model and an IoT-based system were opted for sensing and analysis of type 2 diabetes patients by utilizing type 2 neutrosophic and Višekriterijumsko Kompromisno Rangiranje (VIKOR) methods. The results demonstrated the success of the proposed method showing 9.8% execution time reduction and patient's increased survival rates depending upon symptoms and personal data. Nevertheless, with research, the prediction accuracy could be improved by delving more into advanced machine learning methods. For instance, Yang and coworkers^187^ presented an emotion-alert system by engaging an edge cloud and a personal robot. This system engages users by collecting audio/video data, EEG data, physiological signals and touch data through smart clothing. The system's performance was checked by conducting several tests *via* a specially developed testbed. The results revealed improved mental health of people by the proposed emotion-alert system. Similarly, Chen and coworkers suggested a healthcare system based on Elliptic Curve Cryptography (ECC), which uses a data-driven approach and edge cognitive computing to investigate data related to the physical health of users. The experimental results displayed superior user data cognition, enhanced energy efficiency, cost effectivity, great resource cognition, and enhanced survival rates attributed to the system's rationalized approach. However, it can be perceived that existing technology combined with this system may offer better care opportunities.

A biosensor employs potentiostat for power but recently researchers have shifted towards wearable electrochemical biosensors that are self-powered by biofuels^193^ Based on this, Yu *et al.*,^194^ developed a self-powered wearable sensing platform for the detection of sweat with integrated micro-SCs. NiCo_2_O_4_ has been recognized for its utility in electrochemical sensing devices and SCs, owing to its impressive theoretical specific capacitance and outstanding electrochemical catalytic activity.^194,195^ Moreover, a micro-SC based on NiCo_2_O_4_ was developed for the detection of Na^+^, K^+^ and glucose. The resulting sensor exhibited an enhanced sensitivity of 0.5 mA mM^−1^ and a high capacitance of 18.5 mF cm^−2^. It was further connected with wireless transfer technology, in order to display the personal state of perspiration on a smartphone for the on-spot management of personal health.^196^

Additionally, some wearable sensors such as ring or glove-shaped sensors modified with nanomaterials for the detection of small molecules are designed to be worn on hand.^7,197^ For instance, a ring-shaped plastic device was constructed and implanted with three plastic carbon-based electrodes by a 3D printing technology. Au films electrodeposited onto electrode surfaces serve as active materials for glucose analysis. The resultant e-ring was subsequently integrated with a mini potentiostat for transmitting data to a mobile phone directly.^198^ In order for sensing devices to completely adapt to the irregular or soft body surfaces, textile industry has been adept at incorporating sensing elements. The collaboration aims to build soft fabrics with improved electrochemical characteristics.^199^

## 7. Conclusion

The IoT is a vast technological framework that offers numerous benefits across various domains. It has transformative power in bringing convenience and comfort in our daily lives. The IoT healthcare system is an outstanding service application system that individuals can use by wearing body-worn devices to monitor their health. Enabled by IoT technology, this system integrates various devices and sensors facilitating continuous health monitoring and, hence, helps in regulating human health. This allows for the early detection of health issues and enables personalized interventions. The developments in the IoT and nanotechnology have revolutionized data acquisition in real time, especially in areas with limited resources. It holds great promise for addressing health, welfare and safety concerns. It helps in the early diagnosis of various diseases irrespective of place and time. It facilitates users with quick medical assistance and services. Several sensors can be attached with smartphones, wristbands, tattoos, watches, soft lenses and belts to analyze various aspects of the body including glucose level, body movements, blood pressure and heart rate. The data are then transmitted to the concerned physicians for analysis and monitoring. Moreover, specially designed electrochemical sensing systems are capable of identifying hazardous environments containing volatile organic compounds, toxic gases and industrial vapors, enhancing safety and environmental awareness. The designed electrochemical sensors can offer real-time monitoring of contaminants assisting in public safety and a clean environment. Thus, with the capability to seamlessly gather and analyze data, the IoT can promote proactive healthcare management, safety and security.

## Data availability

The authors declare that the data are available in this manuscript in the form of tables and figures.

## Conflicts of interest

The authors declare no conflict of interest regarding the publication of this manuscript.
